# Targeting T regulatory (T_reg_) cells in immunotherapy-resistant cancers

**DOI:** 10.20517/cdr.2023.46

**Published:** 2024-01-12

**Authors:** Pavlina Spiliopoulou, Paramjit Kaur, Tracey Hammett, Giusy Di Conza, Michael Lahn

**Affiliations:** ^1^Department of Drug Development Program, Phase I Unit, Beatson West of Scotland Cancer Center, Glasgow G12 0YN, UK.; ^2^School of Cancer Sciences, University of Glasgow, Glasgow G61 1BD, UK.; ^3^Department of Oncology Clinical Development, iOnctura SA, Geneva 1202, Switzerland.

**Keywords:** Primary and secondary resistance, T regulatory cells, flow cytometry, mass cytometry, hyperprogression

## Abstract

Primary or secondary (i.e., acquired) resistance is a common occurrence in cancer patients and is often associated with high numbers of T regulatory (T_reg_) cells (CD4^+^CD25^+^FOXP3^+^). The approval of ipilimumab and the development of similar pharmacological agents targeting cell surface proteins on T_reg_ cells demonstrates that such intervention may overcome resistance in cancer patients. Hence, the clinical development and subsequent approval of Cytotoxic T Lymphocyte Antigen-4 (CTLA-4) targeting agents can serve as a prototype for similar agents. Such new agents aspire to be highly specific and have a reduced toxicity profile while increasing effector T cell function or effector T/T regulatory (T_eff_/T_reg_) ratio. While clinical development with large molecules has shown the greatest advancement, small molecule inhibitors that target immunomodulation are increasingly entering early clinical investigation. These new small molecule inhibitors often target specific intracellular signaling pathways [e.g., phosphoinositide-3-kinase delta (PI3K-δ)] that play an important role in regulating the function of T_reg_ cells. This review will summarize the lessons currently applied to develop novel clinical agents that target T_reg_ cells.

## INTRODUCTION

**Figure 1:**
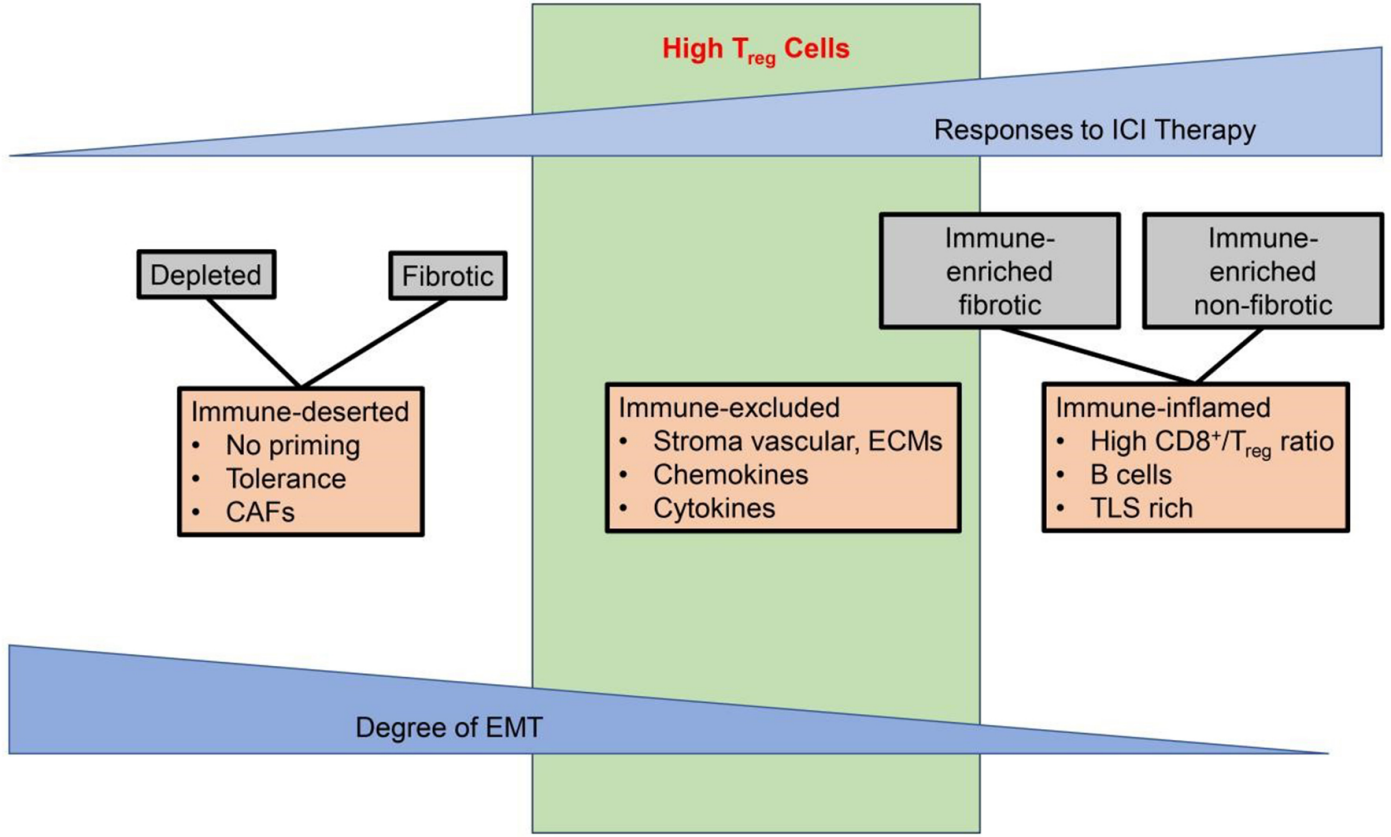
Main Mechanisms of Resistance (primary or secondary) to ICI. There are three different classifications or models summarizing the main mechanisms of resistance to ICI. The first classification (blue triangles) describes the response to ICI in relationship to markers of EMT^[10]^: the more tumors show a status of EMT, the lesser they respond to ICI. The second classification associates the degree and type of fibrosis with responses to ICI (grey boxes)^[8]^: response to ICI is generally observed in conditions with immune-enriched fibrotic and non-fibrotic conditions. By contrast, immune-depleted or fibrotic conditions are not responsive to ICI. The third classification is based on the presence of specific immune cells or markers (red boxes)^[5,6]^: responses to ICI are commonly observed in patients with immune-inflamed conditions (characterized by a high CD8^+^/T_reg_ cell ratio, B cells and TLS-rich tissues); conversely, responses are reduced in immune-excluded conditions (characterized by high vascular stroma content with fibrosis, chemokines, such as CCL, CCL2, CCL5, CCL13, CCL22, or cytokines TGF-β). Limited or no responses to ICI are observed in patients with an immune-deserted tumor microenvironment (lacking T cell priming, exhibiting tolerance, and displaying CAF-related markers). While T_reg_ cells (green box) can be found in each of these conditions, their highest quantity and functional role are observed in either immune-excluded conditions or in immune-enriched fibrotic tissues. ICI: Immune checkpoint inhibitor; EMT: epithelial-mesenchymal transition; TLS: tertiary lymphoid structure; CCL: chemokine c-c-motif ligand; TGF-β: transforming growth factor beta; CAF: cancer-associated fibrosis.

Immunotherapy with immune checkpoint inhibitors (ICI) has become the backbone of several treatment regimens for cancer and has resulted in unprecedented benefits for patients^[1]^. Notwithstanding this progress, many patients eventually experience disease progression while undergoing treatment with ICI, and the mechanisms of the underlying resistance remain elusive^[2]^. One important contributor to such resistance is the immunosuppressive tumor microenvironment^[3-5]^. Based on the state and quality of immune cells, the tumor microenvironment has been classified as immune-inflamed, immune-excluded, and immune-deserted^[6,7]^. A second classification incorporates the role of cancer-associated fibrosis to describe the response to ICI^[8,9]^. A third classification integrates the role of epithelial-mesenchymal transition (EMT) as a key factor for resistance to ICI^[10]^. T regulatory (T_reg_) cells emerge as key contributors of resistance to ICI and are included in each of the three above-mentioned classifications, primarily in immune-excluded or immune-enriched fibrosis conditions [Figure 1]. Considering that T_reg_ cells play an important function in tissue homeostasis, responses to infections, and the control of autoimmunity, their involvement in immune-excluded or immune-enriched fibrosis conditions is perhaps expected^[11]^. Furthermore, T_reg_ cells are no longer recognized as a single group of T cells, but instead consist of different subgroups with varied immunosuppressive properties against which distinct inhibitors can be developed^[12]^. This review will discuss the advances in drug development of large and small molecule agents to overcome T_reg_ cell-mediated resistance to ICI.

## BIOLOGY AND CHARACTERIZATION OF T_reg_ CELLS

### Early discovery of T_reg_ cell biology

Originally described as T suppressor cells^[13-16]^, T_reg_ cells play a specific role in different phases of immune responses^[17]^. T_reg_ cells were first identified as a subset of CD4^+^ T cells by their cell surface expression of CD25 (alpha chain of the IL-2 receptor) and consequently labeled as CD4^+^CD25^+^ T_reg_ cells^[18]^. Functionally, T_reg_ cells were initially characterized by the production of interleukin (IL)-10 and Transforming Growth Factor beta (TGF-β1)^[19]^. Ongoing studies have demonstrated that T_reg_ cells have a high degree of diversity^[17]^. In humans, of all circulating CD4^+^ T cells, approximately 1%-3% are CD4^+^CD25^+^ T_reg_ cells^[20]^. They are often overlooked in clinical studies with respect to their contribution to treatment outcomes of new agents.

### Ontogeny of T_reg_ cells [Figure 2]

**Figure 2:**
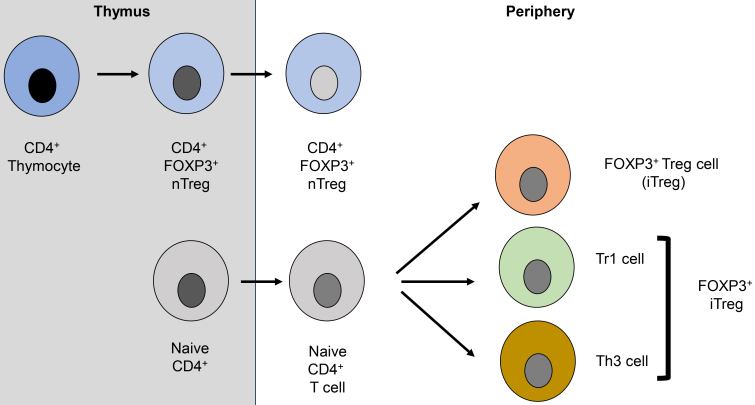
Characterization of T_reg_ cells and subsets: CD4^+^ T cells egress from the thymus and differentiate in blood and tumor tissue. Depending on the degree of CD45RA and FOXP3 expression, CD4^+^ are defined as nT_reg_ cells. CD4^+^ or nT_reg_ cells egress into the periphery, where either cell population is subsequently altered and selected for different types of T_reg_ cells. Based on the “selection model”, CD4^+^ naïve cells are selected to transition into iT_reg_ cells, differing in their functional status as “Tr1 cells” or Th3 cells. FOXP3: Forkhead box protein P3; nT_reg_: natural T regulatory cells; iT_reg_: induced T_reg_ cells; Tr1 cells: type 1 T_reg_ cells; Th3 cells: T helper 3 cells.

T_reg_ cells were defined by their anatomical site of differentiation and the detection of the Forkhead box protein P3 (FOXP3)^[21]^: (1) natural T_reg_ cells (nT_reg_) are T_reg_ cells that develop in the thymus and subsequently migrate to the periphery^[22]^; (2) induced T_reg_ cells (iT_reg_) are those that evolve from naïve CD4^+^FOXP3^-^ T cells upon stimulation in the periphery^[21,23]^. Unfortunately, T_reg_ cells induced *in vitro* were also labeled as iT_reg_ (i.e., inducible T_reg_). This has led to some confusion regarding the nomenclature of T_reg_ cells. Therefore, the 3rd International Conference on regulatory T cells^[24]^ has recommended the following nomenclature to resolve the existing confusion:

1. Thymus-derived T_reg_ cells (tT_reg_) - in lieu of nT_reg_. 2. Peripherally-derived T_reg_ cells (pT_reg_ - i.e., FOXP3^+^ T_reg_ cells that differentiate in the periphery) - in lieu of induced or adaptive T_reg_ cells. 3. *In vitro*-iT_reg_ - i.e., to differentiate T_reg_ cells derived *in vitro* studies from those investigated during *in vivo* studies.

The above-mentioned classifications of T_reg_ cells are based on ontogeny studies and two models are used to describe the generation of T_reg_ cells. The first model is called “instructive model”. According to the “instructive model”, T cells are being “instructed” after T cell receptor (TCR) selection in the thymus. Intermediate TCR stimulation (in contrast to negative and positive selection) leads to the intracellular gene expression of FOXP3, which subsequently determines the generation of T_reg_ cells. The second model is called “selection model”. According to this model, T_reg_ cells are being “selected” rather than “instructed” from a pool of pre-formed T cells. According to this model, *FOXP3* gene expression is independent of the strength of TCR stimulation and further assumes the presence of FOXP3^-^ and FOXP3^+^ T cells in the thymus. Upon exposure to self-antigens, the FOXP3^+^ T cells are resistant to negative selection and form the majority of T_reg_ cells^[25]^.

Independent of the thymus, which is a key organ for the development of T_reg_ cells, secondary lymphoid organs also appear to play a prominent role in generating CD4^+^FOXP3^+^ T cells from CD4^+^FOXP3^-^ T cells^[26]^. Such pT_reg_ cells can originate from sub-immunogenic stimuli, non-inflammatory conditions, long-lasting or chronic infections, and inflammation. Furthermore, they are frequently present in various cancers where they contribute to an immunosuppressive environment^[27-30]^.

### Classification of T_reg_ cells

In general, CD4^+^CD25^+^ T_reg_ cells are characterized by FOXP3^[28-31]^. Additionally, low expression of the IL-7 receptor alpha chain (CD127) on the cell surface of T_reg_ cells often coincides with the intracellular presence of FOXP3^[32]^. Therefore, some classifications use the low expression of CD127 as an alternative marker to FOXP3, recognizing that this may not reflect the entire T_reg_ cell population^[33]^. Using a composite of intracellular and cell surface proteins, four major subsets of CD4^+^ T cells, from which T_reg_ cells are derived, are classified as non-T_reg_, naïve T_reg_, effector T_reg_ and tumor-associated effector T_reg_ cells [Table 1]. Each subset is further characterized by additional surface markers^[31,34]^.

**Table 1:** Two different classifications of T_reg_ cells

**Classification of T_reg_ cells^[31]^**			
**T_reg_ cells subsets**	**Phenotype markers**	**Characteristics**	
Non T_reg_	CD45RA^-^ CD4^+^CD25^+^FOXP3^low^ CTLA-4^+^PD-1^+^	No suppressive activity	
Naïve T_reg_	CD45RA^+^ CD4^+^CD25^+^FOXP3^low^ CTLA-4^low^PD-1^-^	Weak suppressive activity Differentiate into effector T_reg_ cells	
Effector T_reg_	CD45RA^-^ CD4^+^CD25^++^FOXP3^++^ CTLA-4^++^PD-1^+^ GITR^+^LAG3^+^CD127^-^	Strong suppressive activity Prone to apoptosis	
Tumor Effector T_reg_	CD45RA^-^ CD4^+^CD25^++^FOXP3^++^ CTLA-4^+++^PD-1^++^ GITR^++^LAG3^++^CD127^-^	High activation and proliferation	
**Classification of T_reg_ cells based on the concept of “fractions (Fr)”^[34,35]^**			
**Fraction**	**Classification**	**Definition/Phenotype**	**Characteristics**
Fr 1 (= naïve or resting)	rT_reg_	CD45RA^+^ CD4^+^CD25^low^FOXP3^low^ CTLA-4^low^CD127^low/-^Ki67^-^	Derived from the thymus Weak suppressive activity Proliferation and differentiation into effector T_regs_ by TCR stimulation
Fr 2 (= effector or activated)	eT_reg_	CD45RA^-^ CD4^+^CD25^hi^FOXP3^hi^ CTLA-4^hi^, PD-1^+^, ICOS^+^, GITR^+^, OX40^+^, CD15s^+^, CCR4^+^, CCR8^+^, IL-10^+^, TGF-β^+^	Terminal differentiation status Strong suppressive activity Prone to apoptosis Tend to increase in peripheral blood with aging
Fr 3 (= non-T_reg_ cells)	Non-T_reg_	CD45RA^-^ CD4^+^CD25^low^FOXP3^low^ IL-2^+^, IFN-γ^+^, IL-17^+^	Heterogenous population No suppressive activity

T_reg_ cells: T regulatory cells; FOXP3: forkhead box protein P3; CTLA-4: cytotoxic T lymphocyte antigen-4; PD-1: programmed death 1; GITR: glucocorticoid-induced TNFR-related protein; LAG-3: lymphocyte-activation gene 3; TCR: T cell receptor; ICOS: inducible T-cell costimulator; CCR: C-C chemokine receptor; IL: interleukin; TGF-β: transforming growth factor beta; IFN-γ: interferon gamma.

Another nomenclature defines T_reg_ cells as “fractions” [Table 1]^[34,35]^. This nomenclature also takes into consideration elements of functionality. Each T_reg_ cell fraction has distinct functions depending on the type of organ and anatomical location within the organ^[36]^ [Table 1].

Some authors have preferred to define T_reg_ cells based on their function. For example, “type 1 T_reg_ cells” (Tr1) and T Helper (Th)3 cells are T_reg_ cells that produce immunosuppressive factors^[23,37]^. In contrast to the tT_reg_ cells, Tr1 and Th3 T_reg_ secrete the immunosuppressive cytokines IL-10 and TGF-β^[38]^. Others used HELIOS, a member of the Ikaros family of zinc-finger transcription factors, to identify precursors of peripheral T_reg_ cells emerging from the thymus and designated them as nT_reg_^[39]^. Moreover, the expression of neuropilin-1 is used to distinguish T_reg_ cells selected from iT_reg_ in peripheral or extrathymic tissues^[29]^. Recently, the expression of programmed death 1 (PD-1) on T_reg_ cells was found on a highly immune-suppressive subset of T_reg_ cells, especially in patients previously exposed to ICI therapy^[40]^. In summary, these observations underscore the plasticity of T_reg_ cells and the selection of T_reg_ cell subsets in the periphery or extrathymic tissues^[41]^.

### Molecular mechanisms generating T_reg_ cells and their function [Figure 3]

**Figure 3:**
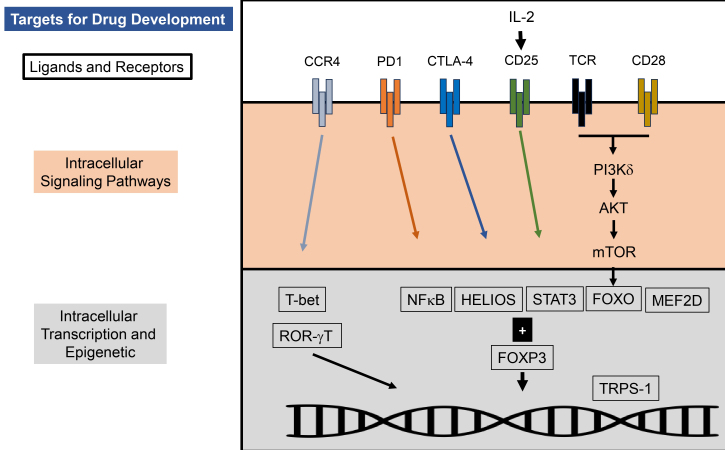
General Concept of Developing Drugs Blocking Activity of T_reg_ cells: In general, there are three main compartments enriched in T_reg_ cells, which are currently being targeted with drugs: (1) Extracellularly by blocking Ligands (white background), such as IL-2. Alternatively, blocking specific receptors on T_reg_ cells, e.g., CTLA-4, CCR4, with monoclonal antibodies, such as ipilimumab or mogamulizumab, can arrest the activity of T_reg_ cells; (2) Intracellularly (red background), signaling pathways can be blocked with small molecule inhibitors, e.g., targeting PI3K-δ; (3) Transcription, gene modification is targeted with different pharmacological agents, such as antisense oligonucleotides, molecular glue, and small molecules. These pharmacological interventions are mainly in non-clinical or early clinical investigations. They target a variety of factors, of which HELIOS and FOXP3 are perhaps the most unique to T_reg_ cells. T_reg_ cells: T regulatory cells; IL: interleukin; CTLA-4: cytotoxic T lymphocyte antigen-4; CCR4: C-C chemokine receptor; PI3K-δ: phosphoinositide-3-kinase delta; FOXP3: forkhead box protein P3.

As highlighted above, FOXP3 is an important intracellular transcription factor determining the fate of T_reg_ cells. The myocyte enhancer factor 2D (MEF2D) is a transcription factor that influences the function of T_reg_ cells^[29,42,43]^. The role of MEF2D is important for two reasons: first, its presence is required for the expression of IL-10, Cytotoxic T Lymphocyte Antigen-4 (CTLA-4), and inducible T-cell costimulator (ICOS) and consequently for the acquisition of the effector T_reg_ cell function. Second, MEF2D acts synergistically with FOXP3^[42]^. Such discoveries point to multiple molecular regulators to generate or maintain T_reg_ cells^[44]^. Consistent with this hypothesis, recent studies have found additional master regulators of human tumor T_reg_ cells^[45]^. By comparing the transcriptional profile of tumor associated with matched peripheral T_reg_ cells from 36 patients with four different malignancies (i.e., glioblastoma, bladder cancer, renal cell carcinoma, prostate adenocarcinoma), 17 master regulators (MRs) were identified^[45]^. *In vivo* CRISPR-cas9 screening with gRNA against these MRs identified Transcriptional Repressor GATA Binding 1 (TRPS-1) as an essential transcription factor for tumor-associated T_reg_ cells. Genetic depletion of TRPS-1 in mice delayed tumor growth by inhibiting infiltration and function of tumor-associated T_reg_ cells, while preserving tolerance in the periphery.

In addition to intracellular transcription factors and the interaction with TCR, chemokines such as C-C motif chemokine ligand (CCL22) can induce the formation of T_reg_ cells^[46]^. CCL22, secreted by dendritic cells (DC) and macrophages, engages with its receptor C-C chemokine receptor (CCR4), which is predominantly expressed on T_reg_ cells^[47]^. Blocking this CCL22/CCR4 axis and consequently removing T_reg_ cells leads to anti-tumor immune responses^[48]^. Recent studies further show that FOXP3 is required to increase the expression of CCR4 on T_reg_ cells^[49]^. This co-regulation underscores that soluble and molecular events determine the fate of T_reg_ cells.

### Epiregulation

The function or the generation of T_reg_ cells can also be influenced by mechanisms of epiregulation^[50]^. In murine models, complement factors determined the methylation of the FOXP3 in T_reg_ cells. Since complement is part of the innate immune system, epigenetic regulation of T_reg_ cells appears to occur early during an immune response. Hence, interventions of blocking complement activation may have an impact on the generation of T_reg_ cells.

### Immunosuppressive function of T_reg_ cells

The classifications of T_reg_ cells can be based on functional studies for all T_reg_ cells or their subsets. Generally, T_reg_ cells exert their suppressive function in three ways: (1) soluble factors; (2) inhibitory receptors; (3) competition for activation or growth factors^[51]^. In recent years, the list of such mechanisms has expanded, and the following examples for each mechanism are presented to illustrate the basis for novel anti-cancer therapies targeting T_reg_ cells.

1. Soluble Factors: IL-10 is secreted by T_reg_ cells and is one of the key cytokines contributing to immune suppression in cancer^[52]^. IL-10 also acts on T_reg_ cells themselves by expanding their number and increasing CTLA-4 expression^[53]^. TGF-β signaling is another cytokine that is associated with immunosuppression by T_reg_ cells^[54,55]^. Like IL-10, TGF-β signaling can also induce T_reg_ cells^[56]^. Its significance might surpass that of IL-10 in the function of T_reg_ cells, as it also inhibits the differentiation and function of Th1 and Th2 cells. TGF-β signaling promotes the differentiation of Th17 and Th9 cells, differentiation of tissue-resident memory CD8^+^ T cells, generation of natural killer (NK) cells, and other tissue-resident cells, e.g., γδ T cells, innate lymphoid cells, and gut intraepithelial lymphocytes^[57]^. Given the tissue distribution of TGF-β signaling proteins and its feedback loop on T_reg_ cells, it may be one factor contributing to the tissue-dependent functionality of T_reg_ cells [Table 2].

**Table 2:** Phenotype characteristics of T_reg_ cells based on tissue distribution highlights the plasticity of T_reg_ cells

**Tissue**	**T_reg_ cell phenotype and function**
Brain	IL-10, IL-33, IL-35, ST2, CTLA-4, TGF-β, IDO, 5-HT_7_, AREG
Lung	COX-2, PGE_2_, TGF-β, AREG, IL-33, CD103, PHD, HIFα
Liver	IL-10, IL-35, CTLA-4, TGF-β, SCFAs, AREG, RA, IDO1, COX2, PGE2, GITR, LAG3, ICOS, CD39/CD73, ST2
Adrenal gland	β1-adrenergic receptors, Glucocorticoid receptor α
Lymph node	IDO, TGF-β, CTLA-4, ICOS, CXCR5, IL-2, CD28, CD103
Skin	IL-10, TGF-β, GITR, CTLA-4, Jag1, IDO, OX40^+^, ARG2, CCR4, CCR6, CLA
Bone	CD39/CD73, RANK, PGE3, TGF-β, IDO, HIF1α, CXCR4

T_reg_ cells: T regulatory cells; IL: interleukin; CTLA-4: cytotoxic T lymphocyte antigen-4; TGF-β: transforming growth factor beta; IDO: indoleamine-pyrrole 2,3-dioxygenase; AREG: amphiregulin; GITR: glucocorticoid-induced TNFR-related protein; LAG3: lymphocyte-activation gene 3; CCR: C-C chemokine receptor.

2. Inhibitory Receptors: Perhaps the most recognized inhibitory receptor expressed on T_reg_ cells is the CTLA-4^[35,58]^. Because of its role in competing with CD28 for the co-stimulatory molecules CD80 (B7.1) and CD86 (B7.2) on antigen presenting cells (APCs), CTLA-4 can induce cell cycle arrest, inhibit the production of IL-2, and down-regulate ligands needed for the activation of T effector cells. Hence, it was termed an immune checkpoint inhibitor (ICI) and this critical discovery was recognized through the Nobel Prize awarded to James Allison and Tasuku Honjo^[59]^. This observation led to the discovery of similar receptors with inhibitory function, such as CD73^[60,61]^. The expression of CD73 in conjunction with TGF-β signaling contributes to a significant increase in T_reg_ cells and renders ICI therapies ineffective.

3. Competition for Growth Factors: Interleukin-2 (IL-2) is not only produced by activated CD4^+^ and CD8^+^ T cells, but also by Dendritic Cells (DCs) and thymic cells^[62]^. IL-2 engages with the IL-2R, which consists of IL-2Rα (=CD25), IL-2Rβ and common γ-chain^[62]^. T_reg_ cells express CD25 constitutively in contrast to T effector cells^[63,64]^. Persistent IL-2 signaling is needed to sustain the T_reg_ cell inhibitory function and survival^[65]^. Insulin Growth Factor was found to act synergistically with IL-2 to achieve persistent T_reg_ cell activity, which suggests that pro-inflammatory conditions support T_reg_ cells^[66]^. Other pro-inflammatory conditions are observed in patients with glioblastoma after receiving a single administration of a Chimeric Antigen Receptor T cell (CAR-T) directed against Epithelial Growth Factor Receptor III^[67]^. After the administration of the CAR-T in patients with glioblastoma, an increase of T_reg_ cells in the tumor microenvironment was observed, which was associated with a lack of treatment response. In another study, children receiving an IL13 CAR-T intracranially showed no reduction in T_reg_ cells in their cerebrospinal fluid^[68]^. Other soluble drivers may originate from metabolic pathways. For example, the fatty acid transporter CD36 sustains mitochondria fitness and the suppressive function of T_reg_ cells in the tumor microenvironment^[69]^. Therefore, T_reg_ cells may not only be influenced by soluble factors, such as cytokines or chemokines, but indirectly affected by factors from the metabolic pathways embedded in the microenvironment.

Overall, these few examples demonstrate that T_reg_ cell function can be induced and maintained by a variety of factors. Hence, activating or blocking these functions is relevant to therapeutic drug development. To appropriately assess the responses to therapies directed against T_reg_ cells, it is necessary to detect and monitor the T_reg_ cells in either tumor tissue or peripheral blood. This assumes that most T_reg_ cells are selected in the periphery and that, regardless of their ontogeny, they share similar mechanisms of action.

## METHODS TO MEASURE T_reg_ CELLS

There are several methods to determine T_reg_ cells in cancer patients. Multiparametric cellular flow cytometry (FC) was historically used to evaluate the T_reg_ cells and their subsets^[70,71]^. Even today, the main advantage of flow cytometry is the quick turn-around time (i.e., generally within hours), and thus can be used to monitor T_reg_ cells before and after novel treatments. An alternative tool to monitor T_reg_ cells is mass cytometry^[72,73]^. Mass cytometry has a reduced risk of signal spill-over, thus improving background noise, and is a highly dimensional method to assess several complex markers simultaneously. The disadvantage of mass cytometry lies in the longer turn-around time, destruction of the specimen at the end of the examination, and the subsequent bioinformatic analyses of high-volume data^[74]^. The power of mass cytometry to measure small subsets of immune cells in blood is exemplified in an ongoing clinical study with the phosphoinositide-3-kinase delta (PI3K-δ) inhibitor roginolisib (IOA-244). In this study, mass cytometry detected a reduction in blood T_reg_ cells across dose cohorts, which was only marginally detected with standard FC^[75]^.

In tumor specimens, standard immunohistochemistry has also provided early insights into changes in T_reg_ cells before and after treatment with standard or novel therapies^[76-78]^. Multiplex immunohistochemistry using a wide range of fluorochromes has increased the ability to simultaneously assess T_reg_ cells and their interaction with adjacent cells, such as CD8^+^ T cells^[79]^. Like standard immunohistochemistry, multiplex studies retain the anatomical features of the specimen and the spatial relationship of cells and stroma, for example, the interaction of T_reg_ cells with APC, CD8^+^ T cells, or tumor cells^[80]^.

Transcriptomics provides another high-dimensional approach to assess T_reg_ cells along with other changes in the tumor or blood^[81]^. Gene expression profiles can describe the T_reg_ cells along with other immune cells using whole tissue extracts^[82]^. Under such conditions, the anatomical structure is lost for the benefit of detecting low signal events. A modification of this technique is single-cell transcriptomics approaches, which have revealed new functions of T_reg_ cells^[83]^. Using this technology, the destruction of the tumor specimen is kept to a minimum while the detection of cellular events is increased. The disadvantage of this technology primarily lies in the processing and evaluation of high-volume data, which leads to long turn-around times.

Like Transcriptomics, Proteomics is a collection of high-dimensional data of proteins either within tumor tissue or proteins shed from tumors to the blood^[84,85]^. Thus, a wide range of secreted proteins can be evaluated, including chemokines (e.g., CCL22) or cytokines (e.g., IL-2, TGF-β) associated with T_reg_ cells^[86]^. For drug development, Proteomics offers a broad discovery tool to study the effect of novel agents. From this discovery platform, specific diagnostic tools can also be developed, such as companion diagnostics or laboratory developed tests.

*In vivo* imaging has been used to describe the dynamics of T_reg_ cells in animals^[87]^. While such studies in animals have shown important insights into T cell regulation in the presence of CTLA-4 inhibition, there are no such specific imaging tools available for appropriate clinical investigation. The most advanced imaging tool uses CD8-labeled PET imaging and reveals significant heterogeneity in CD8^+^ T cell distribution during immunotherapy in patients^[88]^. Therefore, to date, such imaging tools still need to prove their value to guide the drug development of novel agents.

While there are no regulatory-approved tests for assessing T_reg_ cells or their function, FC is the most widely used laboratory test in clinical studies. In contrast to tissue-based tests, T_reg_ cells in the blood can be monitored longitudinally either alone or in comparison to other blood-based immune cells.

## T_reg_ CELLS DURING IMMUNOTHERAPY AND THEIR ROLE IN RESISTANCE

### Background

T_reg_ cells play an important role in tissue homeostasis and co-regulation of other immune cell subsets^[89]^. In the following section, the role of T_reg_ cells during immunotherapy will be reviewed and their potential as either prognostic (i.e., relevant to the disease progression and independent of therapies) or predictive (i.e., in assessing possible response to therapies) biomarkers^[90]^.

### Baseline levels of T_reg_ cells in malignancies and their potential role as prognostic marker [Table 3]

**Table 3:** Examples of malignancies with elevated T_reg_ cells associated with treatment resistance

**Indication**	**Number of patients**	**Method and panel**	**Clinical observation**	**Ref.**
**T_reg_ cells at baseline**				
Pan-cancer	15,512	Meta-analysis of studies assessing FOXP3 in tumor tissue and OS	Influence factors for prognosis included tumor location, molecular subtype, tumor stage For most solid tumors, T_reg_ cells correlated with poor OS	Shang *et al.* 2015^[91]^
Endometrial cancer	82	Flow cytometry using CD4^+^CD25^+^CD127^-^	Baseline associated with treatment resistance	Li *et al.* 2019^[92]^
Endometrial cancer	275	IHC with FOXP3 Flow cytometry using CD4^+^CD25^+^CD127^-^	Tumor tissue enriched for T_reg_ cells at baseline and associated with poor OS Endometrial cancer cells expanded CD4^+^CD25^+^CD127^-^ cells *ex vivo*	Kolben *et al.* 2022^[93]^
Breast cancer	164	Flow cytometry using CD4^+^CD25^+^FOXP3^+^	High T_reg_ cells in tumor tissue and draining lymph nodes associated with invasiveness Associated with CCL5 and increased expression of CCR5 on T_reg_ cells	Qiu *et al.* 2022^[94]^
NSCLC	64	IHC CD3 and FOXP3	High T_reg_ cells in tumor tissue of patients with stage I are at risk of relapse	Petersen *et al.* 2006^[95]^
NSCLC	28	Peripheral blood and flow cytometry using CD4^+^CD25^+^	CD4^+^CD25^+^ is higher compared to healthy subjects Increased CD8^+^CD28^-^ lymphocytes	Karagöz *et al.* 2010^[96]^
NSCLC	23	Peripheral blood and flow cytometry using CD4^+^CD25^+^FOXP3^+^	T_reg_ cells elevated compared to healthy subjects T_reg_ cells increase depending on the stage of NSCLC High intracellular CTLA-4 expression	Erfani *et al.* 2012^[97]^
NSCLC	36	Peripheral blood and flow cytometry using CD4^+^CD25^+^FOXP3^+^	T_reg_ cells elevated compared to healthy subjects T_reg_ cells were negatively correlated with serum IL-17	Hu *et al.* 2018^[98]^
NSCLC	26	Peripheral blood and flow cytometry using CD4^+^CD25^+^FOXP3^+^	T_reg_ cells elevated compared to healthy subjects Correlation of Th17 cells with T_reg_ cells High levels of TGF-β, IL-17, IL-23	Li *et al.* 2014^[99]^
NSCLC	49	Peripheral blood and flow cytometry using CD4^+^CD25^+^FOXP3^+^	T_reg_ cells increase depending on the stage of NSCLC T_reg_ cells decreased after surgery	Chen *et al.* 2014^[100]^
NSCLC	156	Peripheral blood and flow cytometry using CD4^+^CD25^+^FOXP3^+^	T_reg_ cells produce TGF-β and IL-10 Naïve T_reg_ cells elevated and correlated with poor outcome High frequency of terminal T_reg_ cells correlated with improved outcome	Kotsakis *et al.* 2016^[101]^
NSCLC (EGFR mutation)	323 (164 with EGFR mutation)	IHC for FOXP3 (clone 236A/E7)	Significant High FOXP3 expression in EGFR mutation-positive NSCLC Association with poor survival	Luo *et al.* 2021^[102]^
NSCLC (EGFR mutation)	19 (6 EGFR-mutated and 13 EGFR-wildtype)	Flow cytometry with CD45RA^-^FOXP3^+^CD4^+^ (=Fraction 2)	EGFR mutation is non-inflamed (no presence of CD8^+^ T cells) High presence of T_reg_ cells EGFR mutation induces CCL22, which induces T_reg_ cells	Sugiyama *et al.* 2020^[103]^
**T_reg_ cells response during treatment (possible predictive value)**				
Cutaneous melanoma	40	Flow cytometry using CD4^+^CD25^high^CD127^-^Foxp3^+^	High baseline levels Reduction after 3 consecutive doses of ipilimumab Enrichment of CD39^+^HELIOS^+^ T_reg_ cells	Bjoern *et al.* 2016^[104]^
Cutaneous melanoma	32	Flow cytometry using CD4^+^CD25^+^CD127^-^PD-1^+^	Reduction after nivolumab or pembrolizumab treatment observed in patients responding to PD-1 inhibitors No reduction observed in patients with no response	Gambichler *et al.* 2020^[105]^
NSCLC	31	IHC using FOXP3 for tumor tissue and flow cytometry using CD4^+^CD25^+^ FOXP3^+^ for blood	Neo-adjuvant treatment with cetuximab/docetaxel/cisplatin showed a correlation of reduction in T_reg_ cells and response T_reg_ cells at diagnosis did not predict clinical response with therapy	Pircher *et al.*^[106]^
NSCLC	132	Flow cytometry using CD4^+^CD25^+^CD45RA^-^FOXP3^+^	High T_reg_ cells and TGF-β1 levels after 1 week of treatment with PD-1 inhibitors are associated with increased OS High T_reg_ cells at baseline associated with longer OS and PFS	Koh *et al.* 2020^[107]^
NSCLC	27	IHC and mass cytometry T cell subsets	Ratio of PD1^+^ on CD8^+^/PD1^+^ on T_reg_ cells was predictive of outcomes Ratio was predictive in other tumor types as well, i.e., gastric cancer and melanoma	Kumagai *et al.* 2022^[108]^
Renal cell carcinoma	43	Flow cytometry using CD4^+^CD25^+^CD127^-^FOXP3^+^	Treatment with nivolumab reduced T_reg_ cells only in responders when assessed after 3 months Inhibition with CXCR4 antagonist blocked T_reg_ cell function *in vitro* Treatment with nivolumab reduced T_reg_ cells only in responders when assessed after 3 months	Santagata *et al.* 2020^[109]^
Uveal melanoma	9	Mass cytometry using CD4^+^CD25^+^CD127^-^	T_reg_ cells reduced within 3 months, while CD8^+^ and NK cells increased	Di Giacomo *et al.* 2022^[110]^

T_reg_ cells: T regulatory cells; FOXP3: forkhead box protein P3; OS: overall survival; IHC: immunohistochemistry; CCL: chemokine c-c-motif ligand; CCR: C-C chemokine receptor; NSCLC: non-small cell lung cancer; CTLA-4: cytotoxic T lymphocyte antigen-4; IL: interleukin; Th17 cells: T helper 17 cells; TGF-β: transforming growth factor beta; EGFR: epidermal growth factor receptor; PD-1: programmed death 1; NK: natural killer.

The prognostic value of T_reg_ cells was examined by a systematic meta-analysis using data from 76 articles, which included 17 different types of cancers and 15,512 cancer cases^[91]^. This study evaluated T_reg_ cells as part of tumor-infiltrating lymphocytes (TILs). High numbers of T_reg_ cells were associated with shorter overall survival (OS) in most tumor types (e.g., cervical, renal, melanoma, and breast cancer), but were associated with longer OS in colorectal, head and neck, and esophageal cancer. The main parameters that influenced the prognostic value included tumor location, stage of disease, and molecular subtype.

In addition to this meta-analysis, studies assessed the prognostic role of T_reg_ cells in specific tumor types and a few important examples are described below.

In Non-small Cell Lung Cancer (NSCLC), the frequency of T_reg_ cells in peripheral blood increases with the stage of NSCLC^[96,97]^. In 156 NSCLC patients, naïve T_reg_ cells and not terminal T_reg_ cells were correlated with poor outcomes^[101]^. These naïve T_reg_ cells produced TGF-β and IL-10, indicating an immunosuppressive function. A study in the perioperative setting also found that T_reg_ cells in peripheral blood increased with the stage of disease^[100]^. This increase in T_reg_ cells was independent of histology such as squamous and adenocarcinoma. The postoperative T_reg_ cell frequency was not reduced to levels comparable to healthy subjects, suggesting that the immunosuppressive condition remained intact after surgery. Therefore, some investigators proposed to use the presence of T_reg_ cells in tumor tissue to assess the risk for relapse. For example, the T_reg_/TIL Combination Risk Index identified that patients with Stage I NSCLC and a high count of T_reg_ cells were at risk of relapsing^[95]^.

While another study also reported that T_reg_ cells increased with the stage of NSCLC, it found that serum levels of IL-17 and not IL-10 were negatively correlated with T_reg_ cells^[98]^. Gene expression of IL17 in lymphocytes was correlated with numbers of circulating T_reg_, suggesting that IL-17 is being produced by lymphocytes^[99]^. Thus, serum levels of immunomodulatory factors may not always reflect the function of T_reg_ cells in patients. Consequently, for NSCLC patients receiving PD-1 therapies, counts of T_reg_ cells need to be combined with functional assays^[111]^.

In 275 tumor specimens from patients with endometrial cancer, high FOXP3 expression was correlated with poor OS^[93]^. A similar observation was reported for patients with primary breast cancer, where T_reg_ cells and CCL5 were co-expressed with standard prognostic markers for breast cancer^[94]^. The authors postulated that CCL5 engages the CCR5 on T_reg_ cells and subsequently induces the production of TGF-β^[94]^. Like the CCL5/CCR5 axis, the chemokine receptor CCR8 (its ligand being CCL1) also plays a critical role in upregulating genes of intra-tumoral T_reg_ cells as observed in patients with breast, colorectal, and lung cancer^[112,113]^. In each of these tumor types, the expression of CCR8 correlated with T_reg_ cell signature and was associated with poor prognosis^[114]^.

Oncogenic driver mutations are associated with a tumor microenvironment rich in immunosuppressive mediators and T_reg_ cells. For instance, Epidermal Growth Factor Receptor (EGFR) mutations in NSCLC are associated with high levels of T_reg_ cells^[102]^. The microenvironment of patients with EGFR-mutated NSCLC is immune-suppressed, as indicated by tissue expression of FOXP3 and PD-L1^[102]^. Furthermore, high numbers of Fraction 2 T_reg_ cells, low numbers of CD8^+^ T cells (i.e., non-inflamed condition), and high levels of CCL22 (the main ligand for CCR4) are observed in EGFR-mutated NSCLC patients^[103]^. This immunosuppressive state was reversed during combination treatment of EGFR inhibitors and PD-1 monoclonal antibodies, leading to a reprogramming of the immune subsets, and consequently overcoming the resistance. Kirsten Rat Sarcoma Virus (KRAS) mutated tumors are also associated with high numbers of T_reg_ cells, for example, in KRAS-mutated colorectal cancers^[115]^. KRAS-mutated tumors produce the immune suppressive mediators IL-10 and TGF-β1 and thus drive a phenotype switch from naïve to T_reg_ cells^[116]^. Because of these observations in EGFR- and KRAS-mutated tumors, it is possible that other mutations are associated with similar immunosuppressive mediators and T_reg_ cells^[117]^.

In contrast to solid tumors, lymphoma patients may harbor four functionally distinct T_reg_ cell groups: (1) Suppressor T_reg_ cells: similar to solid tumors, this group of T_reg_ cells is immunosuppressive; (2) Malignant T_reg_ cells: the malignant clone derived from precursors of T cells expresses FOXP3 as a marker for adult T cell leukemia/lymphoma (ATLL) and cutaneous T-cell lymphomas (CTCL); (3) Direct tumor-killing T_reg_ cells: T_reg_ cells with suppressive cytotoxicity capable of killing tumor cells; (4) Incompetent T_reg_ cells: mostly observed in angioimmunoblastic T-Cell lymphoma (AITL), and their presence is associated with autoimmune symptoms^[118]^. These different groups with distinct functions were not considered in a recent meta-analysis of 23 lymphoma studies. In this meta-analysis, high numbers of T_reg_ cells at baseline were associated with improved survival^[119]^. However, in some subsets of T cell lymphoma and follicular lymphoma, the high T_reg_ cell counts were not associated with improved OS. Hence, additional differentiation markers are needed to accurately assess the functional role of T_reg_ cells in lymphoma and its sub-types.

While the above-mentioned examples show how T_reg_ cells are associated with survival, it remains unclear whether the presence of T_reg_ cells is merely an epiphenomenon or a key driver of immune suppression in cancer patients. Therefore, changes in T_reg_ cells after clinically meaningful responses to therapies may help to recognize where T_reg_ cells are key drivers of tumor progression.

### T_reg_ cells as potential drivers of tumor progression and their potential role as predictive biomarkers [Table 3]

Studies of immunotherapy and other anti-cancer treatments were selected to determine whether T_reg_ cells are potentially related to treatment outcomes, either as a negative or positive predictive marker^[120]^. For example, patients with hyperprogression during immunotherapy have elevated T_reg_ cells, which is associated with treatment failure^[121,122]^. In such patients, T_reg_ cells expand and copious amounts of immune suppressive cytokines (e.g., TGF-β1, IL-10) are secreted. Furthermore, T_reg_ cells upregulate PD-1 expression during PD-1/PD-L1-targeting therapies, generating highly immunosuppressive T_reg_ cells^[123]^. This observation is not limited to peripheral blood T_reg_ cells. PD-1 expression on T_reg_ cells is also observed in the tumor microenvironment of patients with NSCLC^[108]^. While the expression of PD1 on T_reg_ cells is already predictive for PD-1-based therapies, the ratio of PD1^+^ T_reg_ cells and CD8^+^ T effector (T_eff_) has a superior predictive value than PDL-1 staining alone^[108]^. Hence, detecting PD1^+^ T_reg_ cells by either FC in blood or IHC in tissue can predict the efficacy of ICI therapies.

T_reg_ cell dynamics are not always associated with poor outcomes. For example, PD-L1-treated patients with NSCLC had high frequencies of circulating T_reg_ cells one week after therapy. These levels were correlated with a high response rate, longer progression-free survival, and overall survival^[107]^. At the same time, TGF-β levels were elevated and associated with a favorable response to anti-PD-1 immunotherapy. A second study in patients with cutaneous melanoma also reported an association of high levels of T_reg_ cells with improved outcomes after adjuvant PD-1-based therapies^[124]^. Several reasons may explain this difference between T_reg_ cells as a predictive marker of poor or improved outcomes. First, the mere phenotypic description of T_reg_ cells may ignore certain functional characteristics of T_reg_ cells, which can miss the degree of immune suppression. For instance, T_reg_ cells expressing signal transducer and activator of transcription 3 (STAT3) appear to be less immune suppressive^[124]^. By adding a STAT3 inhibitor to such T_reg_ cells, their suppressive function was enhanced^[124]^. Hence, it is possible that studies reporting increased T_reg_ cells are capturing a broader T_reg_ cell population, including T_reg_ cells, with reduced immunosuppressive function. Second, levels of T_reg_ cells may differ between early and later stages of immunotherapy. Most studies assessed the levels of T_reg_ cells several weeks after starting immunotherapies. Patients with renal cell carcinoma (RCC) treated with nivolumab had a reduction in peripheral T_reg_ cells once they were treated for 3 months, indicating a response to the therapy^[109]^. Similarly, patients with cutaneous melanoma had a significant reduction in T_reg_ cells after three consecutive doses of ipilimumab^[104]^. In uveal melanoma, the peripheral T_reg_ cell population began to decrease after approximately 2 months of treatment with the PI3K-δ inhibitor roginolisib^[110]^. Patients with endometrial cancer who did not respond to immunotherapy had increased T_reg_ cells after several treatment cycles in their blood, indicating a treatment failure^[92]^. Given these differences, it is important to characterize the T_reg_ cell population during a novel therapy before drawing a conclusion on whether T_reg_ cells can serve as a prediction marker. Third, an increase in T_reg_ cells early in therapy may represent a mobilization of the T_reg_ cells from the tumor tissue into the periphery and consequently have limited value for a prediction. Using *in vitro* co-cultures of peripheral blood mononuclear cells (PBMCs) from healthy volunteers, adding them to endometrial cancer cell lines led to an increase of T_reg_ cells within a few hours, suggesting a prompt migratory response of T_reg_ cells^[93]^. Hence, it is possible that once tumor cells are prevented from producing chemoattractant factors as a result of therapeutic intervention, T_reg_ cells may migrate away from the tumor tissue and subsequently be detected in peripheral blood. As mentioned previously, a numerical increase in T_reg_ cells needs to be accompanied by appropriate functional tests to determine whether a change is clinically meaningful.

In hematologic malignancies, T_reg_ cells play a role in the regulation of bone marrow progenitor cells, in controlling the development of malignant clones (e.g., either by transcriptional changes in the malignant B- or T cell), and in influencing the immune cell composition. Some examples are used to illustrate the complexity of targeting T_reg_ cells in hematologic malignancies. Patients with chronic lymphocytic leukemia (CLL) and responding to PI3K inhibitors idelalisib or duvelisib show a reduction in T_reg_ cells^[125]^. Interestingly, this reduction in T_reg_ cells seemed to coincide with toxicities reminiscent of autoimmune toxicities observed in patients receiving ICI^[126]^. Therefore, treatments with oral PI3K-δ inhibitors have offered new insights into the role of T_reg_ cells or their mediators, such as the underappreciated role of IL-17^[127-129]^. Whether this effect of PI3K-δ inhibitors is uniquely related to the reduction in T_reg_ cells remains to be determined, because a reduction or inhibition of the function of T_reg_ cells is not always associated with autoimmune toxicities. One example of T_reg_ cell reduction without autoimmune toxicities is observed in patients receiving Janus kinase (JAK) 1/2 inhibitors in Primary Myelofibrosis (PMF). Patients who respond to the treatment with the JAK 1/2 inhibitor ruxolitinib show a decrease in T_reg_ cells^[130]^. Interestingly, the highest frequency of T_reg_ cells was observed in patients with the highest allele frequency of the JAK2 V617F mutation. Furthermore, long-term treatment with ruxolitinib was associated with disease control and reduction in T_reg_ cells^[131]^. In contrast to the experience with CTLA-4 targeting agents and PI3K inhibitors, the reduction in T_reg_ cells was not associated with autoimmune toxicities. There are at least two factors that may explain the autoimmune toxicities in patients treated with anti-CTLA-4 antibodies or PI3K-δ inhibitors, while they are absent in patients receiving agents while reducing T_reg_ cells. First, common among both drug groups is the question about specificity and selectivity. For example, monoclonal antibodies with a modified Fc framework have an altered response and perhaps also a reduced autoimmune-toxicity profile^[132,133]^. Additionally, for the designated PI3K-δ inhibitors, such as idelalisib^[134]^, parsaclisib^[135]^ and duvelisib^[136]^, the selectivity profile in humans is less clear. All known PI3K-δ inhibitors are not as selective as originally assumed with some important safety implications as recently evaluated^[137]^. Second, in addition to specificity or high selectivity, the immune competency of patients may play a role. For example, in patients with CLL, the B cell function is disrupted. Hence, it is possible that the reduction in T_reg_ cells induces the elevation of cytotoxic Th17 T cells^[125]^.

### Examples of drugs targeting T_reg_ cells and T_reg_ cell-mediated resistance [Table 4]

**Table 4:** Examples of drugs targeting T_reg_ cells

**Drug/Intervention**	**Observation**	**Ref.**
Large molecules		
CTLA-4 targeting agents	Intra-tumoral T_reg_ cells unchanged after ipilimumab or tremelimumab therapy In neo-adjuvant setting, ipilimumab transiently increased T_reg_ cells In patients with metastatic melanoma, T_reg_ cells are reduced after extended treatment time (> 3 months)	Sharma *et al.* 2019^[138]^ Retseck *et al.* 2018^[139]^ Bjoern *et al.* 2016^[104]^ Patel *et al.* 2023^[140]^
PD1 targeting agents	Ratio of expression on T_reg_/T_eff_ cells after immunotherapy potentially predicts response PD1^+^ T_reg_ cells may be dysfunctional	Kumagai *et al.* 2020^[108]^ Lowther *et al.* 2016^[141]^
CCR-4 targeting agents	Monoclonal antibody mogamulizumab (NCT02705105) showed limited activity (ORR or 10%) either as monotherapy or in combination with nivolumab Blood and tumor T_reg_ show a reduction for patients with ORR	Hong *et al.* 2022^[142]^
CCR-8 targeting agents	Subpopulation of T_reg_ cells express CCR-8 Blocking CCR-8 appears not to be associated with autoimmune adverse events in animal studies Monoclonal antibody GS-1811 in early phase clinical trials (NCT05007782)	Kidani *et al.* 2022^[143]^ Weaver *et al.* 2022^[144]^
CD25 targeting agents	CD25 high-affinity subunit alpha Monoclonal antibody RO7296682 (RG6292) had no overt adverse events in animals RO7296682 in clinical trials (NCT04158583)	Solomon *et al.* 2020^[145]^
IL-2 targeting agents	Selective inhibition of trimeric and not dimeric CD25 leads to T_reg_ cell reduction	Wyant *et al.* 2023^[146]^
CEACAM-5 targeting agents	CEACAM-5 and 6 is expressed on highly suppressive T_reg_ cells NEO201 reduces T_reg_ cells	Cole *et al.* 2023^[147]^
Small molecules		
Chemotherapies	Low-dose cyclophosphamide and vaccines Low-dose cyclophosphamide in CRC Docetaxel in NSCLC Sunitinib in RCC	Le *et al.* 2012^[148]^ Ghiringhelli *et al.* 2007^[149]^ Scurr *et al.* 2017^[150]^ Roselli *et al.* 2013^[151]^
STAT3 (FOXP3) inhibition	T_reg_ cell reduction	Revenko *et al.* 2022^[152]^
ATP-competitive PI3K-δ inhibitors	Drug-related Grade 3/4 toxicities limiting continuous dosing and reducing potential efficacy T_reg_ cell reduction in tumor tissue Chemokines inducing T_reg_ cells reduced in lymphoma patients	Eschweiler *et al.* 2022^[153]^ Tarantelli *et al.* 2021^[154]^
Non-ATP competitive PI3K-δ inhibitor roginolisib (IOA-244)	Low grade 3/4 toxicity with no requirement of drug modifications Safety in long-term treated uveal melanoma Reduction in T_reg_ cells, increase in CD8^+^ T and NK cells	Di Giacomo *et al.* 2022^[110]^
JAK1/2 inhibitors	Reduction in T_reg_ cells in patients with PMF responding to ruxolitinib	Massa *et al.* 2014^[130]^
CDK4/6	Reduction in T_reg_ cells and increase in T_eff_ cells, with a greater reduction in patients with responses to therapy	Scirocchi *et al.* 2022^[155]^
BCL2 (e.g., Venetoclax)	Reduction in peripheral T_reg_ cells and enhancement of immune cells	Kohlhapp *et al.* 2021^[156]^

T_reg_ cells: T regulatory cells; CTLA-4: cytotoxic T lymphocyte antigen-4; CCR: C-C chemokine receptor; NSCLC: non-small cell lung cancer; RCC: renal cell carcinoma; STAT3: signal transducer and activator of transcription 3; FOXP3: forkhead box protein P3; PI3K-δ: phosphoinositide-3-kinase delta; NK: natural killer; JAK: Janus kinase; PMF: primary myelofibrosis.

The success of the CTLA-4 targeting agents such as ipilimumab has provided important lessons for future drug development concepts. Herein, we review drug candidates with specific inhibition profiles for T_reg_ cells. Furthermore, the novel agents intend to provide a greater benefit/risk profile. Drugs designed to increase the T_reg_ cells, such as for improving transplantation outcomes, will not be reviewed.

The lessons from the drug development of such agents support the hypothesis that T_reg_ cells are key players in the resistance mechanisms of immunotherapy^[157]^. This explains the increasing number of drug candidates targeting T_reg_ cells with an aim to rebalance the overall immune cell compartment^[12,158]^.

Large Molecules: Because of the preferential expression of CTLA-4 on T_reg_ cells, CTLA-4 inhibitors, such as ipilimumab or tremelimumab, are perhaps the prototype of selective T_reg_ cell inhibitors, although a reduction in T_reg_ cells cannot always be detected^[138-140,159]^. Both ipilimumab and tremelimumab have received approvals for a wide range of indications and form the backbone of many standard treatments^[160]^. With a greater understanding of dose and dose schedule, the use of CTLA-4 targeting agents is evolving. For example, it appears that continuous dosing may not be required to achieve the full effect of CTLA-4 targeting agents^[104,161,162]^. This is best observed in the neo-adjuvant setting, where limited doses of ipilimumab have contributed to a greater disease-free survival and revolutionized treatment for high-risk melanoma patients^[163]^.

In addition to the approved anti-CTLA-4 agents, the group of approved anti-PD-1 targeting agents, such as pembrolizumab and nivolumab, can reduce T_reg_ cells. In contrast to CTLA-4, PD1 is not preferentially expressed on T_reg_ cells. Therefore, the ratio of PD1 expressing T_eff_ and T_reg_ cells can be used as a monitor for response^[108,164,165]^. Whether the PD1^+^ T_reg_ cells are functionally immunosuppressive or have reduced functional activity remains a topic of ongoing research^[141,165]^. Since both main groups of ICI (i.e., CTLA-4 and PD1 targeting agents) affect T_reg_ cells, clinical studies evaluating T_reg_ cells during ICI therapies may provide valuable information for the development of novel inhibitors of T_reg_ cells.

The chemokine receptor CCR4 is expressed on T_reg_ cells. For example, the monoclonal antibody against CCR4, mogamulizumab, is approved for relapsed or refractory mycosis fungoides (MF) or Sézary syndrome (SS)^[166]^. Although mogamulizumab achieved an ORR of 10% in a diverse population with solid tumors^[142]^, T_reg_ cells in tumor tissue and circulating blood were reduced in patients with tumor responses, while there were no changes or even increases in T_reg_ cells for patients who progressed.

In addition to the above-mentioned approved monoclonal antibodies, there are several drug development candidates designed to target specific proteins on T_reg_ cells. One such drug is GS-1811, a monoclonal antibody blocking CCR8 on T_reg_ cells^[143,144]^. This antibody is designed to remove the highly immune suppressive T_reg_ cells, which express CCR8. This approach of reducing a specific subset of T_reg_ cells may address the toxicity concerns otherwise observed with the CTLA-4 targeting agents. Furthermore, it appears that the expression of CCR8 is highly restricted to tumor-infiltrating T_reg_ cells^[144]^.

Targeting CD25 on T_reg_ cells is another selective approach to block T_reg_ cells. RO7296682 (also known as RG6292), a monoclonal antibody designed to specifically block the CD25-mediated function on T_reg_ cells, is currently under clinical investigation (NCT04158583)^[145]^. Due to its design, RO7296682 promises to be more selective and less toxic than prior anti-CD25 monoclonal antibodies, such as daclizumab or basiliximab. As with GS-1811, the anticipated benefit is the reduced toxicity profile compared to the approved CTLA-4 targeting monoclonal antibodies.

Early non-clinical and clinical development efforts are currently targeting the ligand of CD25. This approach relies on blocking IL-2 or modifying the binding of IL-2. Recent technologies can generate multivalent, asymmetric IL-2-Fc fusions with different binding properties (including variable forms to either block or activate T_reg_ cells)^[167]^. A more traditional approach consists in the generation of specific IL-2 blocking antibodies, such as AU-007^[146]^. AU-007 binds to the CD25-binding epitope of IL-2, which prevents the interaction with the trimeric IL-2R expressed on T_reg_ cells, while not affecting the dimer of the IL-2R on memory or naïve T and NK cells. Patients receiving AU-007 had a decrease in T_reg_ cells, with an increase in CD8^+^ T cells. This approach may overcome the known drug resistance in triple-negative breast cancer, where CD25^+^ T_reg_ cells are associated with resistance to immunotherapy^[168]^.

The surface protein CD38 is present on a wide range of immune cells, including T_reg_ cells. The reduction in T_reg_ cells following dosing of the anti-CD38 monoclonal antibody isatuximab plus atezolizumab in patients with advanced solid tumors was evaluated^[169]^. Surprisingly, isatuximab plus atezolizumab was not associated with a reduction in T_reg_ cells, although nearly all patients showed a reduction in CD38^+^ T cells. The low overall response rate, diverse patient population, and low immune cell population at baseline may explain the lack of detectable changes in T_reg_ cells.

The carcinoembryonic antigen-related cell adhesion molecules (CEACAM)-5 and CEACAM-6 are expressed on tumor cells and T_reg_ cells with a profound immunosuppressive function^[170]^. The monoclonal antibody NEO20, which targets CAECAM-5 and -6, reduced T_reg_ cells only in patients with long-term stable disease (SD)^[147]^. Therefore, the observations from the early clinical trials with the anti-CD38 and anti-CEACAM-5 monoclonal antibodies suggest that factors other than selectivity are important in the design of novel T_reg_ cell inhibitors.

Small Molecules: In addition to the large molecules, small molecules are being used to target signaling pathways uniquely or preferentially present in T_reg_ cells. There is an increasing list of small molecules that have been associated with the regulation of T_reg_ cells^[171]^. Perhaps the most common treatments associated with a reduction in T_reg_ cells are chemotherapies, such as cyclophosphamide, either as a therapy alone or in combination with vaccines^[148]^. In particular, the low dose cyclophosphamide (50 mg twice a day for a 2-week of a 4-week cycle) is associated with a reduction in T_reg_ cells and an increase in T_eff_ cells^[150]^. A variation of this administration is the metronomic regimen which also generates reproducible changes in T_reg_ cells^[149]^. Other chemotherapies with immunomodulatory effects include regimens containing docetaxel in NSCLC^[151,172]^, sunitinib in renal cell carcinoma^[151]^, and cisplatin plus vinorelbine in breast and lung cancer^[151]^.

Chemotherapies are not sufficiently selective for T_reg_ cells and their subsets. Hence, more specific inhibitors may target unique pathways of T_reg_ cells, such as targeting FOXP3. Recently, a screen from different compounds found potential candidates that would directly degrade FOXP3, such as derivatives of gallic acid^[173]^. AZD8701 is an antisense oligonucleotide (ASO) blocking STAT3 and thus indirectly FOXP3^[152]^. During the Phase 1 study of AZD8701 in combination with durvalumab (NCT00637039), the FOXP3 expression was reduced with a concurrent reduction in T_reg_ cells.

Following the drug development experience of large molecules targeting CCR4, small molecule inhibitors of CCR4 are being investigated in patients^[174]^. For example, CCR4-351 is a small molecule inhibitor of CCR4, which reduces T_reg_ cells in animal and *in vitro* models^[174]^. CCR4 small molecule inhibitors block the migration of T_reg_ cells and therefore keep T_reg_ cells from entering the tumor microenvironment^[175]^. Despite a wide range of different CCR4 small molecule inhibitors, their clinical development has not led to an approved agent to this date^[176]^.

Another approach is blocking signaling pathways downstream of T cell receptors or co-stimulatory molecules. One such pathway is the PI3K-δ signaling pathway^[177]^. By blocking PI3K-δ signaling, T_reg_ cells show reduced proliferation and, in patients’ plasma, chemokines such as CCL2, CCL3, CCL5, and CCL22 are decreased^[125,154]^. In solid tumors, blocking PI3K-δ signaling modulated immune homeostasis and reinforced PD-1 blockade^[178]^. Based on this observation, the combination of pembrolizumab with parsaclisib (a designated PI3K-δ inhibitor) was investigated in patients who had progressed on prior immunotherapies^[179]^. Unlike the combination of pembrolizumab with the JAK1 inhibitor itacitinib, parsaclisib rebalanced the immune environment towards an interferon (IFN)-γ signature. Patients receiving the combination of parsaclisib and pembrolizumab also showed responses in both ICI-naïve and ICI therapy-resistant tumors (8/28 patients; 28%). Another designated PI3K-δ inhibitor, AMG-319, was investigated in patients with head and neck cancers^[153]^. In post-treatment biopsies, T_reg_ cells were reduced only in patients who tolerated AMG-319 for approximately 2 weeks, and thus were able to complete their scheduled treatment period. The tumor responses were minor and transient, most likely because the treatment was relatively short. These adenosine triphosphate (ATP)-competitive and designated PI3K-δ inhibitors, such as AMG-319 or idelalisib, have limitations due to their toxicity profile in patients with solid malignancies^[180]^. By contrast, the non-ATP, allosteric modulator and highly selective PI3K-δ inhibitor, roginolisib (IOA-244), has a lower rate of severe toxicity, which allows for treatments lasting greater than 6 months^[75,181,182]^. This well-tolerated profile is associated with a reduction in T_reg_ cells and a simultaneous increase of CD8^+^ T and NK cells^[183]^. In patients with metastatic uveal melanoma, these changes in immune cell composition were associated with longer-than-expected overall survival (median OS of 20.8 compared to historic OS of 7.8 months)^[110]^. Whether roginolisib has the potential to overcome resistance to immunotherapy or prevent disease hyperprogression will be the objective of future investigation.

“Molecular glue” compounds, which are derived from cyclosporin A and FK506, are an emerging class of agents for clinical investigation^[184]^. Targeting IKZF2 (the gene that encodes for the zinc finger protein HELIOS, a member of the Ikaros family of transcription factors), the novel glue degrader NVP-DKY709 (=DKY709) reduces tumor resident and circulating T_reg_ cells^[185]^. Because HELIOS is uniquely expressed in a subset of T_reg_ cells^[39]^, this approach promises a selective depletion of T_reg_ cells. DKY709 has been under clinical investigation in a Phase 1 study since 2019, either as a monotherapy or in combination with the PD1 inhibitors PDR001 (NCT03891953; accessed 3rd December 2023). Results on the biomarker responses are soon to be presented.

Reprogramming of T_reg_ cells provides an additional approach to reduce or alter the function of T_reg_ cells^[186-188]^. One such agent is the MALT1 inhibitor, MPT-0118, which in murine models showed a change in tumor-resident T_reg_ cells while not affecting T_reg_ cells in healthy tissue^[189]^. This approach can reduce the anticipated toxicity associated with global T_reg_ cell inhibition. In the first-in-human dose clinical trial, a low toxicity rate was observed along with some functional re-programming of T_reg_ cells^[190]^.

Lastly, there are a growing number of approved small molecules that seem to affect T_reg_ cells, although they were not specifically designed to target T_reg_ cell pathways. We will highlight a few examples to illustrate such underappreciated drugs and their potential as immunotherapeutics. CDK4/6 inhibitors can reduce T_reg_ cells and improve immune responses in patients with breast cancer^[155]^. Similarly, breast cancer patients treated with trastuzumab, either alone or in combination with chemotherapy, showed a reduction in T_reg_ cells^[191]^. The JAK1/2 inhibitor ruxolitinib is associated with a reduction in T_reg_ cells in patients with primary myelofibrosis^[130,131]^. The FLT3 inhibitor midostaurin reduced T_reg_ cells in PBMCs from patients with AML^[192]^. Whether this effect is mediated via Dendritic Cells is being investigated^[193]^. The BCL2 inhibitor venetoclax, alone and in combination with pembrolizumab, improves immune responses and is associated with the reduction in T_reg_ cells in animal studies^[156]^. SRC inhibition represents another target for T_reg_ cell modification. The SRC inhibitor dasatinib seems to reduce T_reg_ cells and enhance immune responses in preclinical models^[194]^. While these aforementioned approved small molecule inhibitors do not specifically target signaling pathways in T_reg_ cells, they seem to have clinical benefits associated with a reduction in T_reg_ cells. This opens a new avenue for the rapid development of new immunotherapies with established agents as pursued by clinical research initiatives^[195,196]^.

## CONCLUSION

Lessons from the drug development of CTLA-4 inhibitors may provide valuable insights to successfully develop new therapies targeting T_reg_ cells. The research on T_reg_ cells has uncovered a T cell population with great plasticity. Despite their relatively small size, T_reg_ cells play a critical role in modulating immune responses to tumors. Hence, for novel drugs to be successfully developed in the clinic, the appropriate methods to assess the function of T_reg_ cells need to be evaluated alongside the standard measures of clinical benefit. The discovery of the precise pharmacologic platform (i.e., large or small molecule) that will deliver the greatest advantage is currently an exciting area of drug development.

## DECLARATIONS

### Copyright

© The Author(s) 2024.
